# METTL3-mediated m6A RNA methylation induces the differentiation of lung resident mesenchymal stem cells into myofibroblasts via the miR-21/PTEN pathway

**DOI:** 10.1186/s12931-023-02606-z

**Published:** 2023-11-28

**Authors:** Yi Lu, Zeyu Liu, Yunjiao Zhang, Xiuhua Wu, Wei Bian, Shan Shan, Danrong Yang, Tao Ren

**Affiliations:** Department of Respiratory and Clinical Care Medicine, Shanghai Sixth People’s Hospital Affiliated to Shanghai Jiao Tong University School of Medicine, Shanghai, 200233 China

**Keywords:** Lung resident mesenchymal stem cells (LR-MSCs), M6A methylation, Myofibroblast, METTL3/miR-21/PTEN pathway, Pulmonary fibrosis (PF)

## Abstract

**Background:**

The accumulation of myofibroblasts is the key pathological feature of pulmonary fibrosis (PF). Aberrant differentiation of lung-resident mesenchymal stem cells (LR-MSCs) has been identified as a critical source of myofibroblasts, but the molecular mechanisms underlying this process remain largely unknown. In recent years, N6-methyladenosine (m6A) RNA modification has been implicated in fibrosis development across diverse organs; however, its specific role in promoting the differentiation of LR-MSCs into myofibroblasts in PF is not well defined.

**Methods:**

In this study, we examined the levels of m6A RNA methylation and the expression of its regulatory enzymes in both TGF-β1-treated LR-MSCs and fibrotic mouse lung tissues. The downstream target genes of m6A and their related pathways were identified according to a literature review, bioinformatic analysis and experimental verification. We also assessed the expression levels of myofibroblast markers in treated LR-MSCs and confirmed the involvement of the above-described pathway in the aberrant differentiation direction of LR-MSCs under TGF-β1 stimulation by overexpressing or knocking down key genes within the pathway.

**Results:**

Our results revealed that METTL3-mediated m6A RNA methylation was significantly upregulated in both TGF-β1-treated LR-MSCs and fibrotic mouse lung tissues. This process directly led to the aberrant differentiation of LR-MSCs into myofibroblasts by targeting the miR-21/PTEN pathway. Moreover, inhibition of METTL3 or miR-21 and overexpression of PTEN could rescue this abnormal differentiation.

**Conclusion:**

Our study demonstrated that m6A RNA methylation induced aberrant LR-MSC differentiation into myofibroblasts via the METTL3/miR-21/PTEN signaling pathway. We indicated a novel mechanism to promote PF progression. Targeting METTL3-mediated m6A RNA methylation and its downstream targets may present innovative therapeutic approaches for the prevention and treatment of PF.

**Supplementary Information:**

The online version contains supplementary material available at 10.1186/s12931-023-02606-z.

## Background

Pulmonary fibrosis (PF) is an irreversible interstitial lung disease characterized by decreased lung function and progressive dyspnea [1, 2]. Most PF patients experience rapid clinical deterioration with a median survival of only 2 to 4 years and eventually die from respiratory failure [3]. Although some antifibrotic drugs, such as pirfenidone and nintedanib, can delay PF progression in part, there is still no effective treatment for fibrosis reversal to date [4]. Thus, understanding the novel pathogenesis of PF is critical to developing more effective therapeutic approaches against PF.

Aberrant myofibroblast activity has been recognized as a central factor in PF pathogenesis. Studies have found that myofibroblasts are primarily derived from endogenous precursor/stem cells, which can rapidly proliferate and differentiate into myofibroblasts after tissue injury [5, 6]. Investigating the mechanism of endogenous lung stem cell differentiation into myofibroblasts during the pathological progression of PF may contribute to generating novel therapeutic strategies to prevent or reverse fibrosis.

Lung resident mesenchymal stem cells (LR-MSCs) are a subpopulation of endogenous lung stem cells with pluripotent differentiation potential that actively participate in the development of intrapulmonary pathology [7]. LR-MSCs share the same morphological, immunophenotypic, and multilineage differentiation ability as bone marrow-derived mesenchymal stem cells [8]. Research has established that LR-MSCs are integral to preserving the natural structure and proper functioning of the lungs, which can differentiate into adipogenic, osteogenic and chondrogenic lineages, thus promoting tissue repair. Aside from their reparative capabilities, LR-MSCs can modulate intrapulmonary pathology under specific circumstances, as they are highly sensitive to their surrounding microenvironment. For patients with different chronic lung diseases, including PF, COPD, and bronchiectasis, LR-MSCs shared similar phenotypes as those from healthy individuals but had different differentiation directions [9]. We previously reported that LR-MSCs could differentiate into myofibroblasts in a TGF-β1-inducible fibrosis model [10]. LR-MSCs from bronchiolitis obliterans patients are more likely to differentiate into myofibroblasts than normal cells [11]. Similarly, another study revealed that both the abundance and myofibroblast differentiation potential of LR-MSCs were significantly elevated in an allergic asthma model [12]. Overall, aberrant differentiation directions of LR-MSCs form the foundation of numerous diseases, including PF, but the specific mechanism remains poorly characterized.

N6-methyladenosine (m6A) RNA methylation is among the most widespread posttranscriptional modifications, constituting nearly 50% of all methylated ribonucleotides [13, 14]. Its widespread presence and dynamic nature make it a pivotal regulator of several cellular processes, such as export, splicing, translation, and turnover [15]. Dysregulation of m6A has been linked to various diseases, including cancer, neurological disorders, and fibrosis. m6A writers, including methyltransferase-like 3 (METTL3), methyltransferase-like 14 (METTL14), and Wilms tumor 1-associated protein (WTAP), have been reported to promote fibrosis by enhancing the mRNA stability of profibrotic genes [15]. Previous studies have shown that m6A RNA methylation is intimately linked to fibrosis progression in diverse fibrotic disorders, including cardiac fibrosis, liver fibrosis, and renal fibrosis [16–18]. However, the precise function of m6A RNA methylation in the occurrence and development of PF is still poorly understood.

Our study aimed to explore the involvement and molecular mechanism of m6A RNA methylation in the differentiation of LR-MSCs into myofibroblasts, which is regarded as an underlying mechanism for PF progression. Our results demonstrated that m6A modification mediated by METTL3 could trigger the myofibroblastic differentiation of LR-MSCs by modulating the miR-21/PTEN pathway. These findings suggest that targeting METTL3-mediated m6A may provide promising therapeutic strategies for PF prevention and treatment.

## Methods

### Ethics statement

This study was carried out under the guidance of the Ethics Committee of Shanghai Sixth People's Hospital (ethics approval no. 2020-YS-096). All experiments involving animals were conducted in strict compliance with IACUC guidelines.

### Isolation, culture, and identification of mouse LR-MSCs

Murine lung parenchyma was cut into 0.5–1 mm pieces and then digested with an enzyme cocktail (Suzhou Junxin Biotechnology Co., Ltd.). LR-MSC populations were initially selected by magnetic anti-CD45 and anti-Sca-1 beads (Miltenyi Biotec, Bergisch Gladbach, Germany). LR-MSCs from the primary screen were then cultured with MSC-specific medium (Suzhou Junxin Biotechnology) and passaged using MSC-specific digestion solution (Suzhou Junxin Biotechnology).

Flow cytometric analysis was performed for cell type identification. Cell surface markers, including CD44, CD73, CD90, CD34, CD45, CD14, and CD19R, were involved in this study. After digestion, LR-MSCs were resuspended and washed three times with PBS and then incubated for 1 h at 37 °C with FITC-labeled antibodies as indicated above (Biolegend, Beijing, China). Cells were analyzed using a FACS CaliburTM flow cytometer (Becton Dickinson).

The multilineage differentiation potential of LR-MSCs toward osteogenic, chondrogenic, and adipogenic cells was further assessed. Adipogenic, osteogenic and chondrogenic differentiation were induced using adipogenic induction solution, osteogenic induction solution and chondrogenesis-inducing fluid, respectively. Oil red staining and alkaline phosphatase activity were positive for lipid accumulation and osteogenesis differentiation. Chondrogenic differentiation potential was confirmed by the observation of chondrogenic nodules. Induction solution and identification kits were purchased from Suzhou Junxin Biotechnology Co., Ltd.

### Modulation of gene expression

First, three specific small hairpin RNAs were synthesized for METTL3 (shMETTL3s) and PTEN (shPTENs) knockout and integrated into the lentiviral vector pLKO.1. Then, total gene synthesis was used to clone the METTL3 and PTEN cDNAs into the lentiviral overexpression pHIV vector. After the lentiviral vector was constructed, viral packaging was carried out in 293 T cells. Finally, viral particles were collected, and LR-MSCs were infected for 72 h to obtain stable cells with corresponding gene overexpression or knockdown.

### Quantitative real-time PCR analysis (qRT‒PCR)

To collect the total RNA of cultured cells or tissues, RNA extraction kits from Takara were utilized, followed by cDNA synthesis using a PrimeScript^™^ RT reagent kit from Takara. qPCR was then performed on the ABI 7500 Fast Real-Time PCR System from Applied Biosystems using a qPCR mix procured from Takara. To measure mRNA and miRNA expression levels, β-Actin and U6 were utilized as reference housekeeping genes. All gene primers for the assay are listed in Additional file 2: Table S1 and were synthesized at Sangon Biotechnology Co., Ltd. (Shanghai, China).

### Western blotting (WB)

Protein lysates from tissues or cultured cells were extracted using RIPA cell lysis buffer (Cell Signaling Technology Inc., Danvers, MA), and their concentration was determined by the BCA assay (Sangon Biotechnology, Shanghai, China). Subsequently, proteins were separated and transferred onto PVDF membranes, and primary antibodies against METTL3, METTL14, WTAP, FTO, ALKBH5, type I collagen, α-smooth muscle actin (α-SMA), vimentin, and β-actin (Abcam, Cambridge, MA) were added and incubated overnight at 4 °C. After washing three times, HRP-conjugated secondary antibodies were added and incubated for 2 h. Immunoreactive signals were measured using the Tanon-4500 gel imaging system (Shanghai, China), with β-Actin serving as an internal control.

### m6A dot blot

m6A dot-blot assays were performed based on a previous study [19]. In short, RNA molecules were denatured by heating them at 65 °C for 5 min and dissolved in 50 µl of 20 × SSC buffer (Sangon Biotech, Shanghai, China). The RNAs were then spotted onto the Amersham Hybond-N + membrane (GE Healthcare) and UV-crosslinked to the membrane. Once washed with 1 × PBST buffer (Sangon Biotech, Shanghai, China), the membrane was blocked with 3% BSA for 1 h. Next, anti-m6A antibodies (1:1000, ab284130, Abcam) were added and incubated overnight at 4 °C. After three washes, HRP-conjugated secondary antibodies were added and incubated for 2 h. Immunoreactive signals were measured using the Tanon-4500 gel imaging system (Shanghai, China), with β-Actin serving as an internal control. To detect consistency among different groups, the membrane was stained using 0.02% methylene blue (MB) in 0.3 M sodium acetate (pH 5.2).

### RNA immunoprecipitation (RIP)

The cultured cells were lysed using immunoprecipitation lysis buffer consisting of 10 mM NaCl, 0.5% NP-40, 200 units/ml RNase inhibitor, EDTA-free cocktail, 10 mM Tris–HCl, and 1 mM DTT. Then, the cell lysate was incubated in METTL3, m6A, DGCR8, or Ago2 antibody along with magnetic beads and lysis buffer at 4 °C overnight. Following this, the protein‒RNA complex was separated using proteinase K, and an RNA extraction kit was utilized to extract the RNA. RNA enrichment was measured by qRT‒PCR analysis.

### Cellular immunofluorescence

Cultured cells were washed with PBS, fixed with paraformaldehyde (4%) and permeabilized with Triton-100 (0.5%). Subsequently, treated cells were incubated with antibodies targeting type I collagen, α-SMA and Vimentin. Next, the cells were incubated with the corresponding secondary antibodies, and the nuclei were stained with Hoechst. After staining, the results were visualized using immunofluorescence microscopy (Olympus, Toyota, Japan).

### m6A messenger RNA methylation prediction

RNA sequences of hsa-miR-21 (pri-miRNA and mature miRNA) were retrieved from miRTarBase (https://mirtarbase.cuhk.edu.cn). m6A messenger RNA methylation prediction was performed by SRAMP. The predictive scores for m6A modification potential were determined through the complete transcript mode and the generic (default) model for tissue selection and categorized as very high, high, moderate, and low scores (cuilab.cn/sramp/). The local RNA structures of potential modification sites were also calculated and visualized.

### Animal experiments

Female C57BL/6 mice (6 weeks old) were procured from Slac Laboratory Animal Corporation (Shanghai, China). A quarantine period of seven days was implemented before the mice were subjected to bleomycin via tracheal injection at a dose of 5 mg/kg. Following continuous feeding for four weeks, the Penh coefficient was calculated via lung scanning to confirm successful model construction. To evaluate the involvement of METTL3 in the progression of PF, adeno-associated viral particles carrying shRNA targeting METTL3 (AAV-shMETTL3) and vector AAV particles (AAV-shNC) were intravenously injected via the tail vein prior to bleomycin administration at a concentration of 1 × 10^9^ AAV particles per mouse. Mice were euthanized through abdominal injection of 10% chloral hydrate overdose (3 ml/kg), and tissue samples were obtained for primary LR-MSC isolation, HE staining, q-RTPCR, and WB experiments. H&E staining was carried out by Service Biotechnology Co., Ltd. (Shanghai, China). Primary LR-MSC isolation, q-RTPCR, and WB experiments were performed as previously described.

### Statistical analysis

The study was conducted using three independent biological replicates unless otherwise stated in the figures. All results are presented as the mean and standard deviation (mean ± SD). Student’s t test was conducted to evaluate the significance of differences between two groups, and one-way ANOVA either with or without Tukey‒Kramer testing was applied to analyze differences among three groups. Statistical significance was defined as P < 0.05 in all analyses.

## Results

### LR-MSCs differentiate into myofibroblasts in response to TGF-β1

**Fig. 1:**
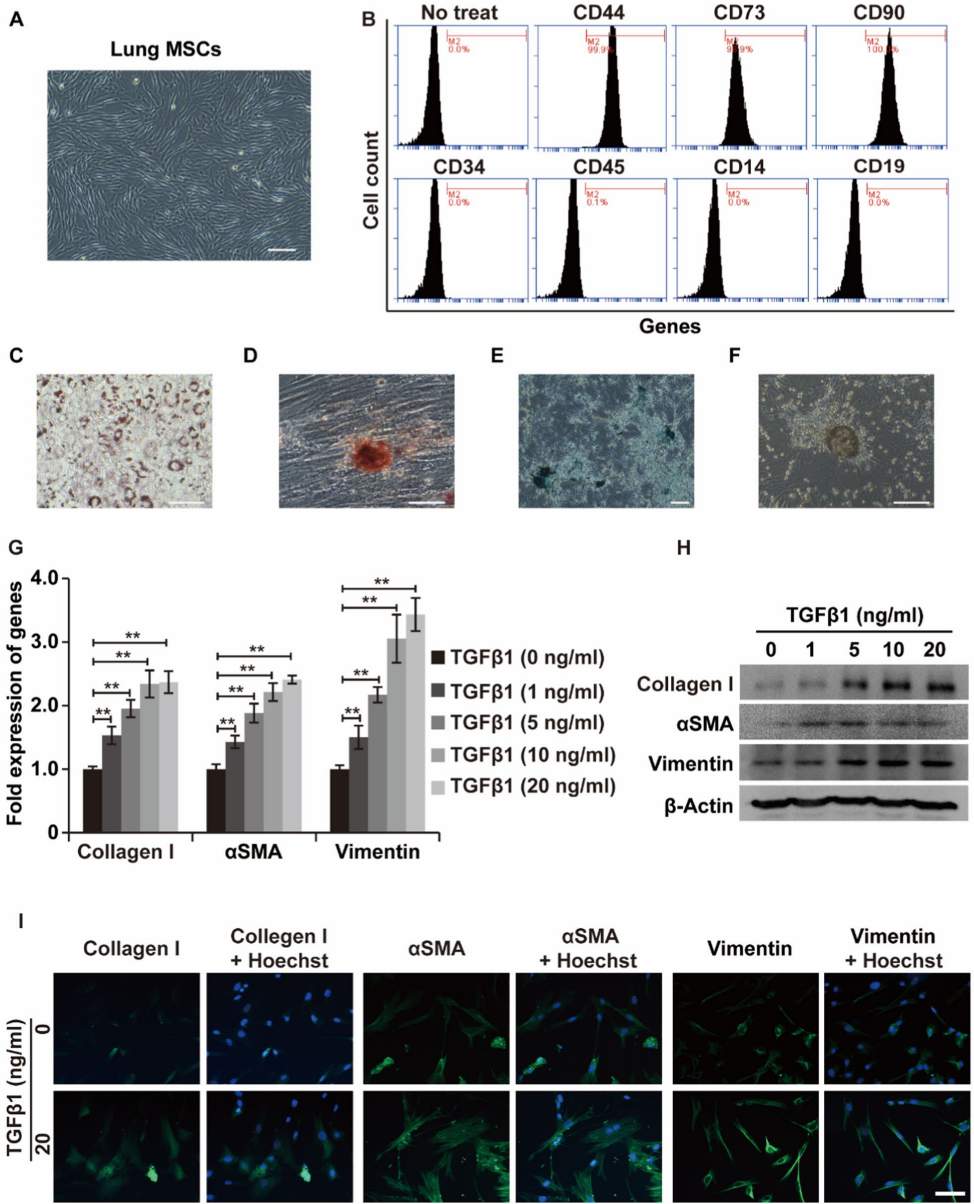
LR-MSCs differentiate into myofibroblasts in response to TGF-β1. **A** Morphologies of mouse LR-MSCs under a standard light microscope. Scale bar: 200 μm. **B** Flow cytometric analysis of surface markers (CD44, CD73, CD90, CD34, CD45, CD14 and CD19R) on LR-MSCs. **C**–**E** Adipogenic (oil red O staining), osteogenic (alizarin red staining) and chondrogenic (alcian blue staining) differentiation potential of LR-MSCs. **F** Cartilage nodules formed by LR-MSCs during chondrogenic differentiation. **G**–**I** Relative changes in the mRNA and protein levels of the indicated markers related to myofibroblast differentiation (α-SMA, type I collagen, and vimentin) in LR-MSCs at baseline and after TGF-β1 stimulation, as assessed by qRT‒PCR, WB assays and immunofluorescence analysis. β-Actin was used as a reference gene. Scale bars = 20 μm, **P < 0.01

LR-MSCs were isolated from mouse lung tissue and showed long-spindle, helical, and radial morphological arrangements when cultured in vitro (Fig. 1A). After expansion, LR-MSCs in culture expressed typical mesenchymal cell surface markers (CD44, CD73, and CD90) and were negative for hematopoietic, endothelial, and monocyte markers (CD34, CD45, CD14, and CD19R) (Fig. 1B). The multidifferentiation capacities of LR-MSCs (adipogenic, osteogenic and chondrogenic) were further confirmed by Oil red O staining, Alizarin red staining, and Alcian blue staining (Fig. 1C–F). Taken together, high-purity MSCs with good differentiation potential were successfully isolated in this study.

TGF-β1 is a crucial cytokine implicated in pulmonary fibrosis (PF) that can regulate cell differentiation programs. In our experiments, LR-MSCs were treated with progressively increasing TGF-β1 concentrations (0, 1, 5, 10, and 20 ng/ml) to mimic the human PF microenvironment. The results indicated that the expression levels of myofibroblast markers (α-SMA, type I collagen, and vimentin) in treated LR-MSCs increased with TGF-β1 concentrations and peaked at the highest concentration (Fig. 1G–I). In summary, these results confirmed that TGF-β1 induced myofibroblastic differentiation of LR-MSCs, which might contribute to PF progression.

### METTL3-mediated m6A RNA methylation is involved in TGF-β1-inducible myofibroblastic differentiation of LR-MSCs

**Fig. 2:**
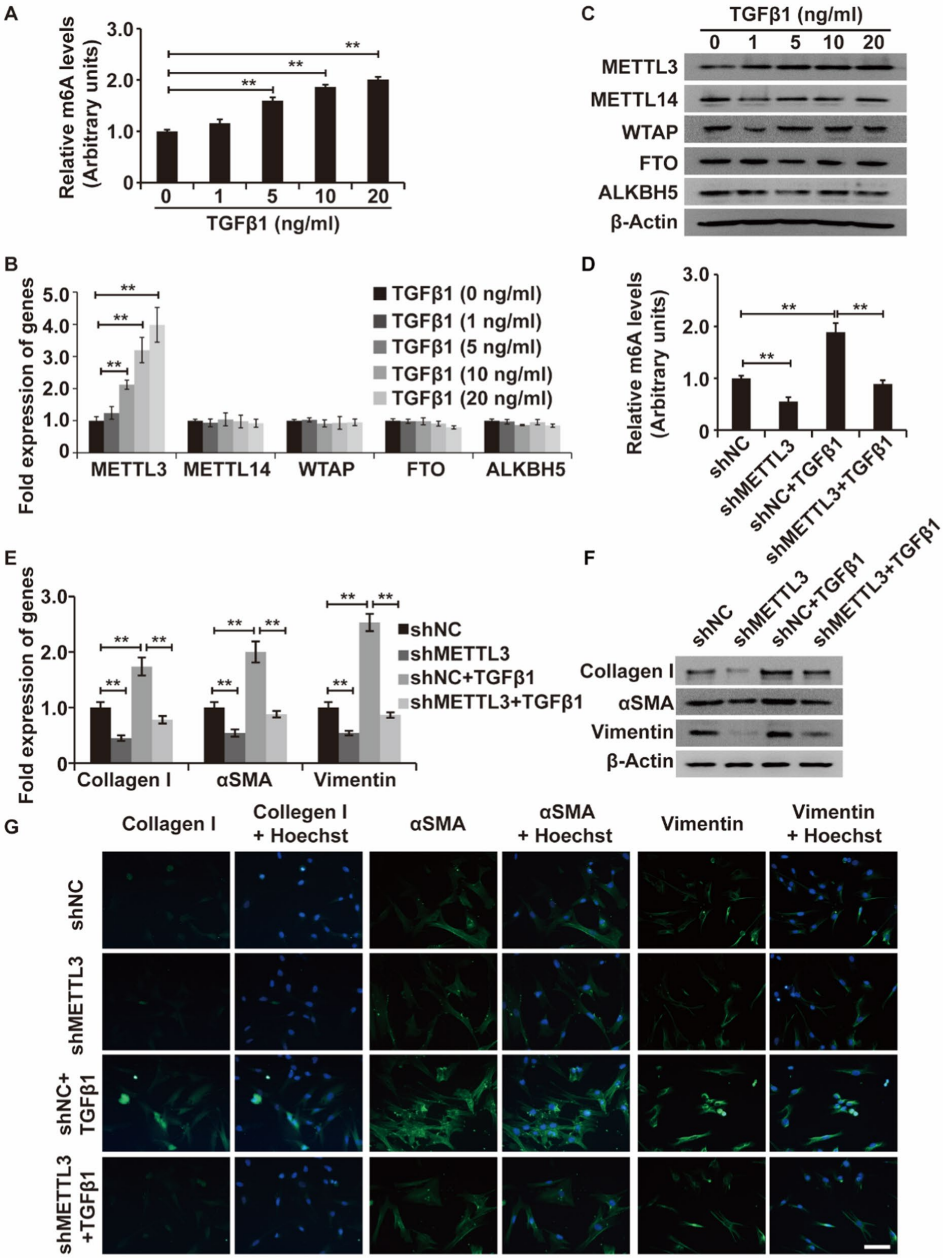
TGF-β1-induced differentiation of LR-MSCs into myofibroblasts is dependent on METTL3-mediated m6A RNA methylation. **A** Relative m6A levels (arbitrary units) of LR-MSCs at baseline and after TGF-β1 stimulation. **B**, **C** The mRNA and protein expression levels of METTL3, METTL14, WTAP, FTO, and ALKBH5 in LR-MSCs at baseline and after TGF-β1 stimulation by qPCR and WB assays. **D** Relative m6A levels (arbitrary units) of LR-MSCs in the following four cases: control group, METTL3 silencing group, TGF-β1-treated group, and METTL3 silencing group treated with TGF-β1. **E**, **G** The mRNA and protein expression levels of myofibroblast markers (α-SMA, type I collagen, and vimentin) in LR-MSCs treated as described above. β-Actin was used as a reference gene. Scale bars = 20 μm, **P < 0.01

m6A RNA methylation is intimately linked to fibrosis progression in diverse fibrotic disorders [16–18]. However, the involvement of m6A RNA methylation in PF has not been investigated. Given the importance of aberrant LR-MSC differentiation in PF progression, we measured the m6A RNA methylation levels in TGF-β1-treated LR-MSCs for the first time. We found that m6A RNA methylation levels tended to increase with increasing TGF-β1 stimulus levels (Fig. 2A), which suggested that the effect of TGF-β1 on the direction of LR-MSC differentiation is associated with m6A RNA methylation.

To explore the detailed mechanism of m6A RNA methylation in TGF-β1-treated LR-MSCs, we measured the components of the main catalytic system of m6A, including METTL3, METTL14, WTAP, FTO, and ALKBH5. We found that METTL3 was remarkably upregulated at the RNA and protein levels under TGF-β1 stimulation, whereas the expression levels of METTL14, WTAP, FTO, and ALKBH5 showed no appreciable changes (Fig. 2B and C). Taken together, these findings indicated that METTL3 played a significant role in regulating m6A modification in TGF-β1-treated LR-MSCs.

We next constructed METTL3-knockdown or METTL3-overexpressing LR-MSCs for cell function experiments. Compared with the corresponding controls (shNC or pHIV-NC), shMETTL3s dominantly repressed METTL3 expression, while pHIV-METTL3 obviously elevated METTL3 expression in LR-MSCs (Additional file 1: Fig. S1). Upon stimulation with TGF-β1, the repression of METTL3 significantly inhibited the TGF-β1-induced elevation of m6A RNA methylation (Fig. 2D) and further reversed the TGF-β1-induced upregulation of type I collagen, α-SMA, and vimentin in LR-MSCs (Fig. 2E–G). In other words, TGF-β1-inducible myofibroblastic differentiation of LR-MSCs was dependent on METTL3-mediated m6A RNA methylation.

**Fig. 3:**
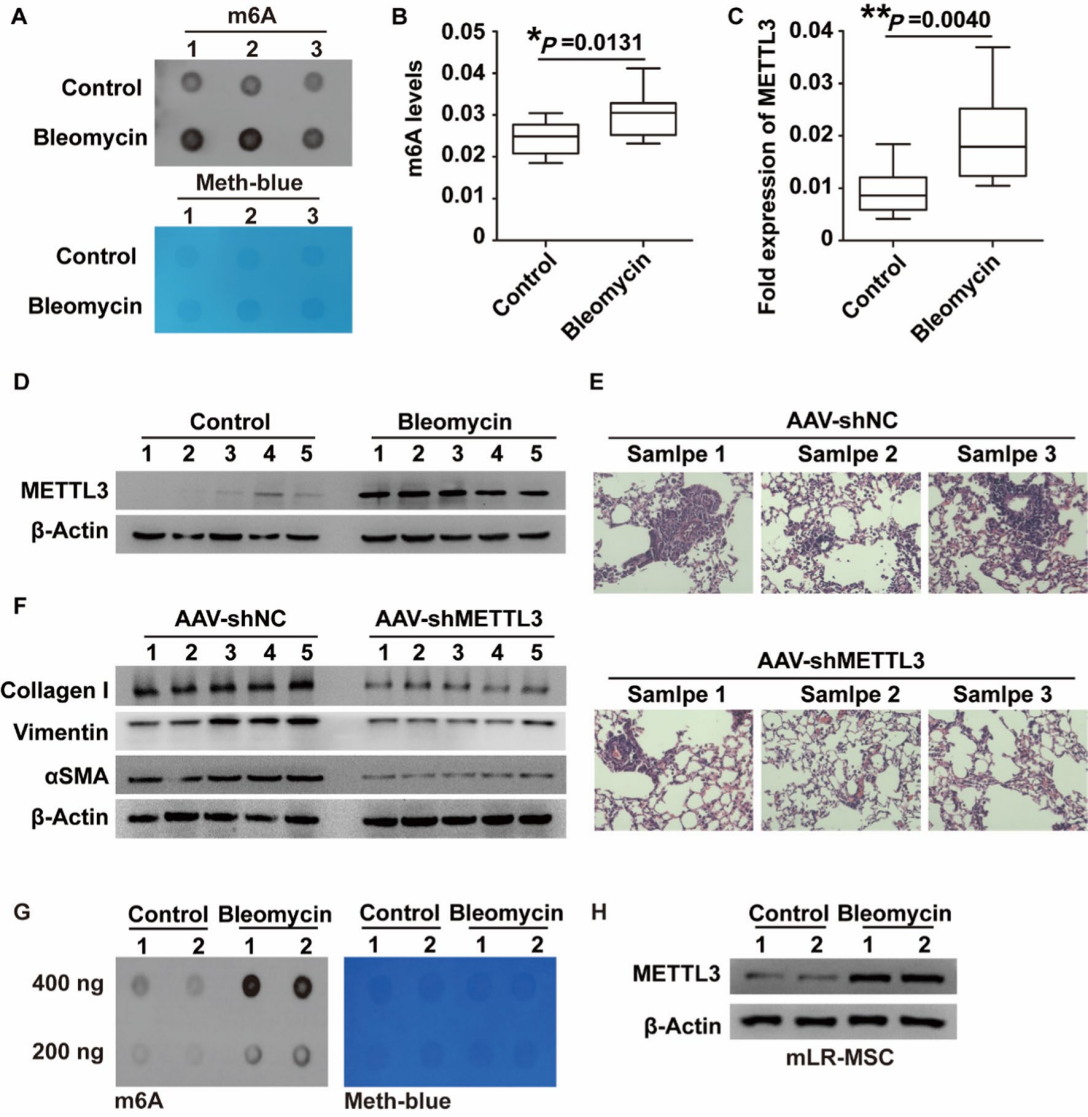
METTL3-mediated m6A RNA methylation is involved in TGF-β1–induced pulmonary fibrosis in the murine lung. **A**, **B** The m6A levels (arbitrary units) in mouse fibrotic lungs relative to normal lungs. **C**, **D** The mRNA and protein expression levels of METTL3 in mouse fibrotic lungs relative to normal lungs by qRT‒PCR and WB assays. **E** HE staining of lung tissues from mice exposed to bleomycin with or without METTL3 silencing. **F** The α-SMA, type I collagen, and Vimentin expression levels in lung tissues from mice exposed to bleomycin with or without METTL3 silencing. β-Actin was used as a reference gene. **G**, **H** The m6A levels and METTL3 levels in primary LR-MSCs isolated from mouse fibrotic lungs and normal lungs. *P < 0.05, **P < 0.01

To further support the above findings, bleomycin-induced PF mouse models were constructed (Additional file 1: Fig. S2). Consistent with the in vitro results, the m6A RNA methylation levels (Fig. 3A, B) and METTL3 expression levels (Fig. 3C, D) were both markedly increased in the lung tissues of PF mice compared with controls. Moreover, when the METTL3 gene was silenced, the fibrosis severity of bleomycin-treated mice was markedly attenuated (Additional file 1: Fig. S3, Fig. 3E, F). Furthermore, we established the m6A RNA methylation levels and METTL3 expression levels in primary LR-MSCs isolated from the mouse lung tissues. The results of western blot assay and dot-blots assay demonstrated that, compared to that in LR-MSCs from the mouse in sham group, both the levels of METTL3 expression and m6A modification were increased in LR-MSCs from the bleomycin-induced fibrosis model (Fig. 3G, H). In summary, METTL3-mediated m6A RNA methylation is involved in PF progression by promoting abnormal LR-MSC differentiation.

### METTL3-mediated m6A RNA methylation promotes miR-21 maturation

Increasing evidence suggests that METTL3-mediated m6A modification marks primary miRNAs (pri-miRNAs) and allows for the effective recognition of the microprocessor complex subunit DGCR8, which in turn modulates pri-miRNA processing and enhances miRNA maturation [20, 21]. Aberrant miRNA levels are associated with several pathologies, including cancer and fibrotic disease. In our study, we analyzed the potential target pri-miRNA of m6A according to previous literature and prediction of m6A modification sites.

**Fig. 4:**
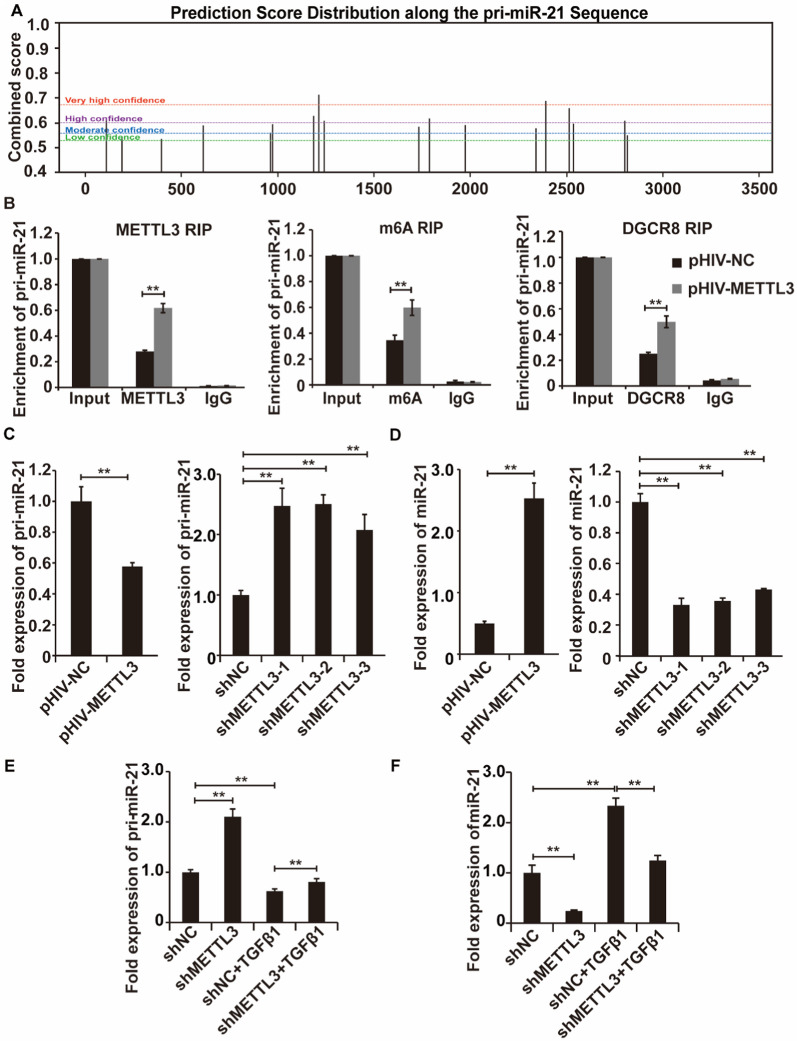
METTL3-mediated m6A RNA methylation promotes pri-miR-21 processing by DGCR8. **A** Prediction score distribution of the potential m6A modification sites along the pri-miR-21 sequence. **B** RIP-qPCR analysis of pri-miR-21 enrichment by METTL3, m6A and DGCR8 in LR-MSCs with or without METTL3 silencing. (C-D) qRT‒PCR analysis of pri-miR-21 (**C**) and miR-21 (**D**) expression levels in the METTL3 overexpression group or METTL3 silencing group compared to the control. **E**, **F** qRT‒PCR analysis of pri-miR-21 (**E**) and miR-21 (**F**) expression levels in the following four cases: control group, METTL3 silencing group, TGF-β1-treated group, and METTL3 silencing group treated with TGF-β1. U6 and β-Actin were used as reference genes. **P < 0.01

miR-21 has been reported to play key roles in diverse organ fibrosis, such as renal fibrosis, intestinal fibrosis, cardiac fibrosis and pulmonary fibrosis[22–25]. Our findings indicated that the pri-miR-21 sequence possessed multiple potential m6A modification sites according to the m6A-modified prediction scores (Fig. 4A). As an example, local RNA structures of pri-miR-21 with very high confidence in m6A modification potential are visualized in Additional file 1: Fig. S4. The GGACA motif, the recognition sequence of METTL3, was highly enriched within the m6A modification sites of pri-miR-21. Accordingly, we speculated that pri-miR-21 was the potential target of METTL3-mediated m6A modification in TGF-β1-treated LR-MSCs. METTL3, m6A and DGCR8 RIP assays were subsequently performed. The results showed that pri-miR-21 was significantly enriched by METTL3, m6A and DGCR8, and the enrichment levels were increased with METTL3 overexpression (Fig. 4B). METTL3-mediated m6A modification promotes pri-miRNA processing to mature miRNA by DGCR8. Thus, pri-miR-21 expression levels were decreased when METTL3 was overexpressed and elevated after knockdown (Fig. 4C), whereas miR-21 levels displayed the opposite trend (Fig. 4D). Overall, pri-miR-21 displayed a significant decreasing trend upon TGF-β1 stimulation and rose considerably when METTL3 was silenced (Fig. 4E), and the trends of miR-21 were in the opposite direction (Fig. 4F). Collectively, these findings indicated that METTL3-mediated m6A modification facilitated miR-21 maturation in TGF-β1-treated LR-MSCs.

### miR-21 mediates TGF-β1-inducible myofibroblastic differentiation of LR-MSCs

**Fig. 5:**
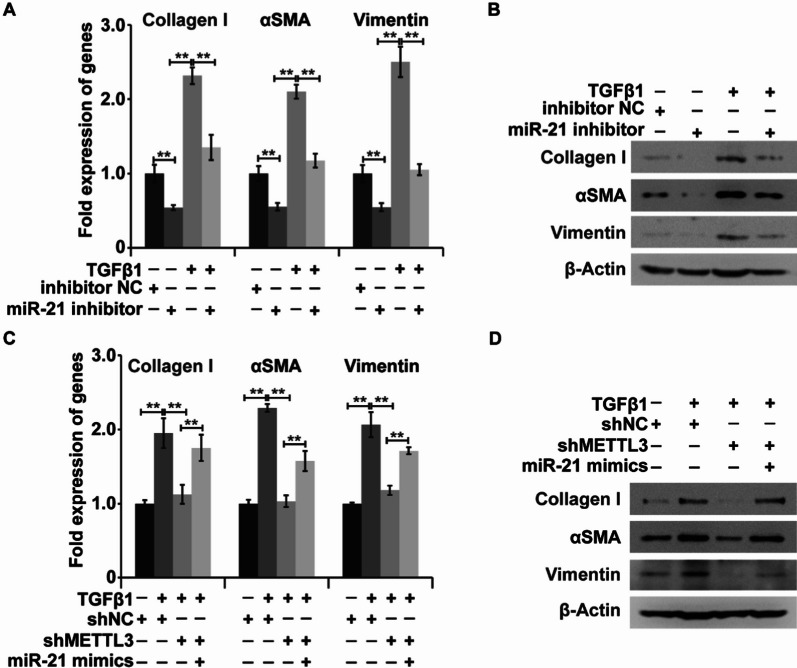
METTL3 is involved in TGF-β1-induced differentiation of LR-MSCs into myofibroblasts by targeting miR-21. **A**, **B** The mRNA and protein expression levels of myofibroblast markers (α-SMA, type I collagen, and Vimentin) in LR-MSCs with or without TGF-β1 treatment and miR-21 inhibitor. **C**, **D** The mRNA and protein expression levels of myofibroblast markers (α-SMA, type I collagen, and Vimentin) in LR-MSCs with or without METTL3 silencing, combined with or without treatment with TGF-β1 and miR-21 inhibitor. β-Actin was used as a reference gene. **P < 0.01

To examine whether miR-21 mediated the TGF-β1-induced myofibroblastic differentiation of LR-MSCs, we treated LR-MSCs with TGF-β1 in the absence of miR-21. The results indicated that miR-21 inhibition could reverse the TGF-β1-inducible upregulation of type I collagen, α-SMA, and vimentin at the RNA and protein levels in LR-MSCs (Fig. 5A and B). We then investigated whether METTL3 is involved in this process by modulating miR-21. From qPCR and WB analyses, we identified that silencing METTL3 markedly reduced the process of TGF-β1-inducible myofibroblastic differentiation, which could be partially reversed by miR-21 mimics (Fig. 5C and D). Collectively, these results support the idea that miR-21 mediated TGF-β1-inducible myofibroblastic differentiation of LR-MSCs, which was dependent on METTL3.

### miR-21 targets PTEN to sustain TGF-β1-inducible myofibroblastic differentiation of LR-MSCs

**Fig. 6:**
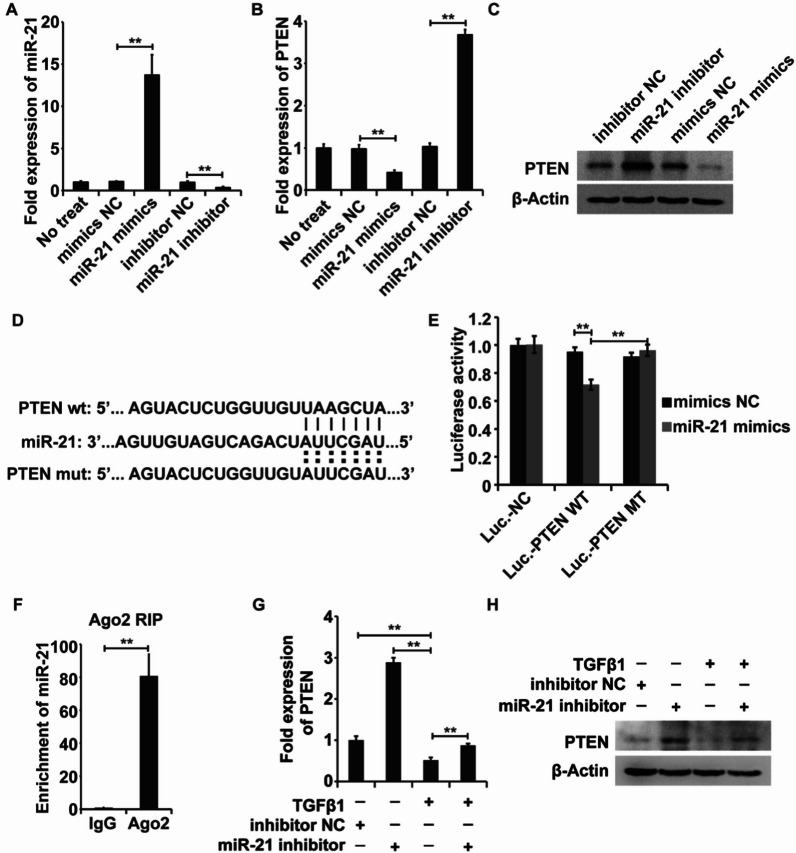
miR-21 inhibits PTEN expression in LR-MSCs. **A** qRT‒PCR analysis of miR-21 expression with the addition of miR-21 inhibitor or mimics in LR-MSCs. **B**, **C** qRT‒PCR and WB analysis of PTEN expression with the addition of miR-21 inhibitor or mimics in LR-MSCs. **D** Construction of the luciferase reporter system. **E** Dual-luciferase reporter assay to validate the direct binding between miR-21 and the PTEN 3′-UTR. **F** RIP-qPCR analysis of miR-21 and its interacting 3′ UTR of PTEN enrichment by Ago2. **G**, **H** The mRNA and protein expression levels of PTEN in LR-MSCs with or without TGF-β1 treatment and miR-21 inhibitor. β-Actin was used as a reference gene. **P < 0.01

We predicted the candidate downstream target genes of miR-21 using TargetScan (http://www.targetscan.org/), miRDB (http://www.mirdb.org/cgi-bin/search.cgi), and miRcode (http://www.mircode.org/). PTEN was identified as a candidate downstream target gene of miR-21. To test this notion, we measured PTEN expression levels in the presence of miR-21 inhibitors, miR-21 mimics, and their control RNAs (inhibitor NCs and mimic NCs). PTEN expression was negatively correlated with miR-21 levels through qPCR and WB assays (Fig. 6A–C). To investigate whether miR-21 directly targeted PTEN, we cloned the 3′-UTR of PTEN mRNA containing either the wild-type or mutant binding site of miR-21 into the luciferase reporter plasmid psiCheck-2 (Luciferse-PTEN-wt or Luciferse-PTEN-mut) (Fig. 6D). The miR-21 mimics potently inhibited the luciferase activity of Luciferse-PTEN-wt but had no impact on Luciferse-PTEN-mut (Fig. 6E). Additionally, miR-21 was enriched in Ago2 immunoprecipitates, which were ternary complexes containing Ago2, miRNAs and their interacting 3’ UTR of PTEN (Fig. 6F). In general, PTEN expression obviously decreased upon TGF-β1 treatment and could be partly restored by miR-21 inhibitors (Fig. 6G and H). Collectively, we demonstrated that PTEN is the downstream target of miR-21 in TGF-β1-treated LR-MSCs.

**Fig. 7:**
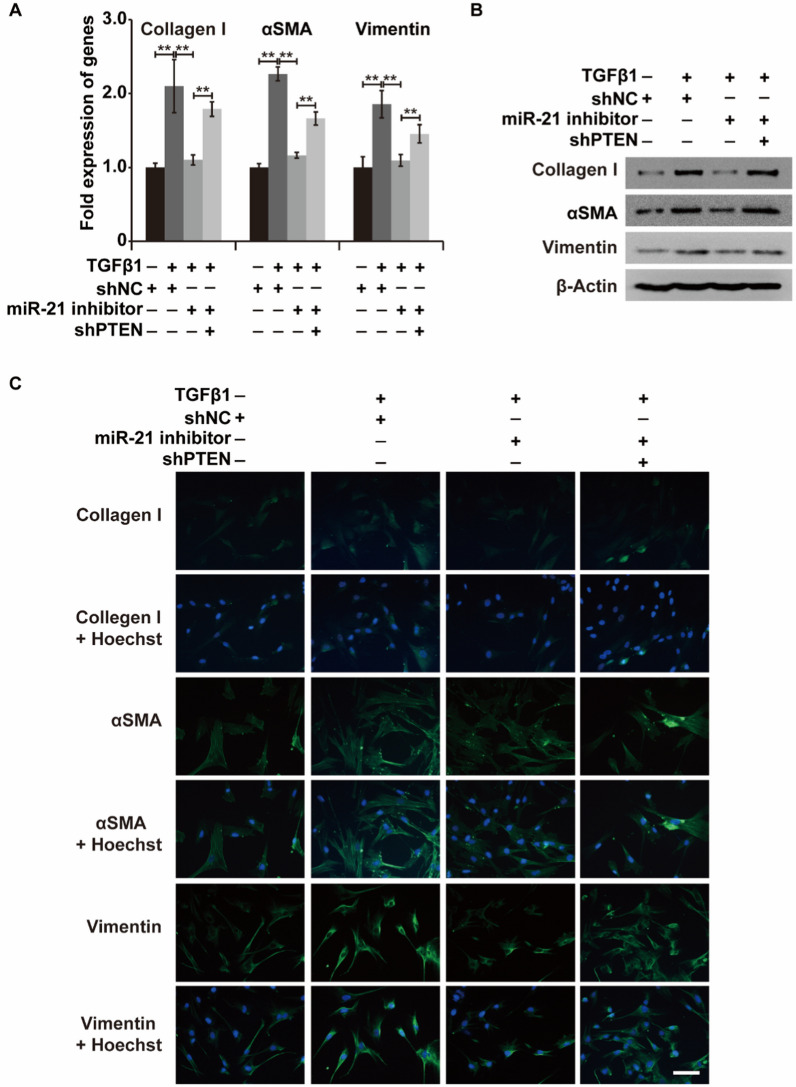
miR-21 affects the TGF-β1-induced differentiation of LR-MSCs into myofibroblasts by regulating PTEN expression. **A**–**C** The mRNA and protein expression levels of myofibroblast markers (α-SMA, type I collagen, and Vimentin) in LR-MSCs with or without PTEN silencing, combined with or without treatment with TGF-β1 and miR-21 inhibitor. β-Actin was used as a reference gene. Scale bars = 20 μm, **P < 0.01

To further assess the role of PTEN in TGF-β1-treated LR-MSCs, we constructed PTEN-knockdown or PTEN-overexpressing LR-MSCs (Additional file 1: Fig. S5). Upon stimulation with TGF-β1, PTEN silencing significantly reversed the miR-21 inhibitor-induced downregulation of type I collagen, α-SMA, and Vimentin in LR-MSCs (Fig. 7A–C). In summary, miR-21 promoted the TGF-β1-inducible myofibroblastic differentiation of LR-MSCs by suppressing PTEN expression.

### TGF-β1-inducible myofibroblastic differentiation of LR-MSCs is mediated by METTL3/miR-21/PTEN pathway

**Fig. 8:**
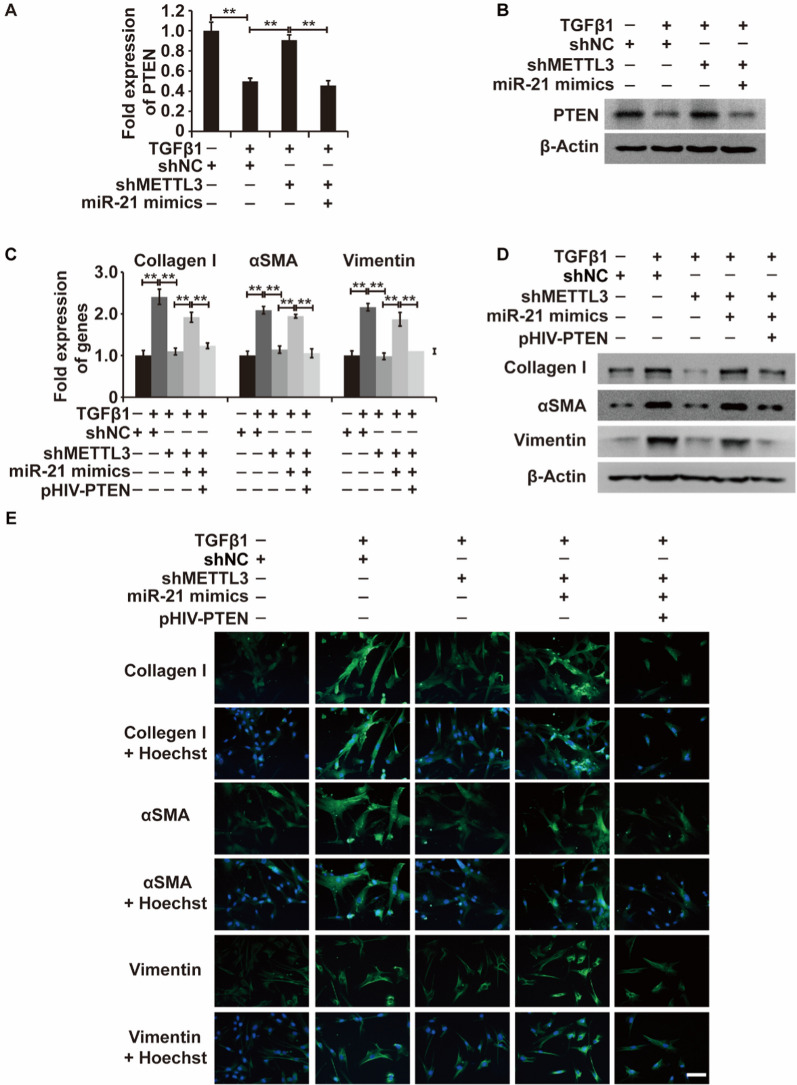
TGF-β1 induces the differentiation of LR-MSCs into myofibroblasts by modulating the METTL3/miR-21/PTEN pathway. **A**, **B** The mRNA and protein expression levels of PTEN in LR-MSCs with or without METTL3 silencing, combined with or without treatment with TGF-β1 and miR-21 mimics. **C**–**E** The mRNA and protein expression levels of myofibroblast markers (α-SMA, type I collagen, and vimentin) in LR-MSCs with or without METTL3 silencing, PTEN overexpression, and treatment with TGF-β1 and miR-21 mimics. β-Actin was used as a reference gene. Scale bars = 20 μm, **P < 0.01

Based on the above findings, we aimed to assess the relationships among METTL3, miR-21, and PTEN in TGF-β1-treated LR-MSCs. Our study found that METTL3 inhibition significantly reversed the TGF-β1-induced decrease in PTEN expression, and these expression levels could be reversed by miR-21 mimic addition (Fig. 8A and B). Thus, the METTL3/miR-21/PTEN regulatory pathway was aberrantly activated in TGF-β1-stimulated LR-MSCs.

We next examined the role of the METTL3/miR-21/PTEN pathway in the process of LR-MSC differentiation into myofibroblasts. We found that any disturbance in the METTL3/miR-21/PTEN pathway was confirmed to impact the progression of differentiation (Fig. 8C–E). Overall, the METTL3/miR-21/PTEN pathway played a key regulatory role in TGF-β1-inducible myofibroblastic differentiation of LR-MSCs.

## Discussion

PF is a fatal, progressive fibrotic lung disorder typified by the accumulation of myofibroblasts and excess deposition of ECM [1]. In our research, we found that LR-MSCs, as a subpopulation of endogenous lung stem cells with pluripotent differentiation potential, are a novel source of myofibroblasts in the presence of TGF-β signaling. This plays a crucial pathological role in PF progression. However, the detailed molecular mechanism of the aberrant differentiation of LR-MSCs into myofibroblasts remains unclear. M6A RNA methylation is a posttranscriptional modification that can impact RNA stability and translation and has been linked to various human diseases [26]. Our work reveals that METTL3-mediated m6A RNA methylation levels can facilitate miR-21 maturation. Increased miR-21 consequently leads to PTEN inhibition and ultimately drives the abnormal differentiation of LR-MSCs into myofibroblasts upon TGF-β1 signaling stimulation.

MSCs are a versatile class of pluripotent stem cells that reside in bone marrow and various organs, such as the lung, liver, and brain, and possess the potential to differentiate into mesenchymal lineages. Evidence suggests that LR-MSCs, which possess the ability to differentiate into adipocytes, osteoblasts, and chondrocytes, contribute to maintaining pulmonary homeostasis and facilitating lung repair [27]. Although these cells seem purely beneficial, they may also contribute to lung disease in certain circumstances [28, 29]. Previous studies have shown that LR-MSCs are involved in PF progression [30]. Inflammatory stimuli in the local microenvironment after injury can promote alveolar epithelial cells to release cytokines or chemokines, such as TGF-β1, during the early stage of PF, which leads to further abnormal wound healing [31, 32]. In our research, we confirmed that LR-MSCs abnormally differentiated into myofibroblasts upon TGF-β1 stimulation, which was consistent with previous studies [33, 34]. Myofibroblasts are known to be involved in dysfunctional matrix remodeling, which can induce chronic lung disease [35]. Our study successfully demonstrates that the differentiation direction of LR-MSCs is sensitive to the microenvironment, which represents a novel mechanism to promote PF progression.

Multiple studies have reported that TGF-β1 interacts with the METTL3-METTL14-WTAP complex via SMAD2/3, connecting m6A RNA methylation to TGF-β1 signaling [36]. Moreover, m6A methylation has been shown to influence the differentiation potential of MSCs [37]. However, little is known about the involvement and detailed mechanism of m6A methylation in LR-MSC differentiation into fibroblasts. In our current investigation, we found that the levels of m6A methylation and the expression of its regulatory enzyme METTL3 were significantly elevated in both TGF-β1-treated LR-MSCs and fibrotic mouse lung tissues. Furthermore, inhibiting METTL3 expression effectively mitigated the TGF-β1-inducible myofibroblastic differentiation of LR-MSCs by decreasing m6A methylation levels. Therefore, METTL3-mediated m6A modification is a promising therapeutic target against PF.

Previous research has indicated that METTL3-mediated m6A methylation has broad effects on mRNA stability and translation efficiency, as well as on miRNA processing and maturation [20]. Pri-miRNAs with m6A modifications are recognized by DGCR8, which selectively binds to specific substrates, thereby promoting miRNA maturation and leading to elevated miRNA expression [38]. Thus, these findings suggest that m6A methylation is a critical regulator of miRNA levels, and the complex interactions between miRNAs and m6A regulatory proteins require continued investigation. In our research, we found that METTL3-mediated m6A methylation promotes pri-miR-21 processing and maturation, thereby driving LR-MSC differentiation into myofibroblasts. These results suggest that the METTL3/miR-21 regulatory pathway is involved in LR-MSC myofibroblastic differentiation. To fully comprehend the function of m6A methylation in the regulation of miR-21 and its role in the onset and progression of PF, more in-depth investigations are needed.

Several studies have reported a negative correlation between miR-21 expression and PTEN levels. The significant effect of miR-21 on PTEN expression suggests their interplay in various biological processes. PTEN is known as a tumor suppressor and has been found to participate in various human cancers. Increasing evidence suggests that PTEN is involved in the pathogenesis of PF [39, 40]. Baosong Xie reported that PTEN levels were obviously reduced in IPF patients, and PTEN overexpression inhibited TGF-β1-inducible fibroblast differentiation into myofibroblasts [41]. Similarly, Jing Geng et al. found that inhibition of USP13-mediated PTEN protein loss caused phenotypic changes in fibroblasts and impacted the pathogenesis of IPF. Two previous studies have demonstrated that the loss of PTEN induces lung fibrosis by activating the NF-κB signaling pathway during the senescence of alveolar epithelial cells [42, 43]. Collectively, these findings imply that PTEN plays a crucial role in the TGF‑β1-inducible differentiation of fibroblasts into myofibroblasts. However, prior research has not focused on the function of PTEN in the differentiation of LR-MSCs into myofibroblasts. Our results demonstrated a decrease in PTEN expression during TGF‑β1-inducible myofibroblastic differentiation of LR-MSCs. Moreover, PTEN silencing significantly affected the role of the METTL3/miR-21 pathway during this differentiation process. These findings suggest a crucial role for PTEN in LR-MSC differentiation into myofibroblasts, providing new insights into the pathogenesis of PF.

Overall, our study indicated a novel mechanism to promote PF progression. We revealed that m6A RNA methylation induced aberrant LR-MSC differentiation into myofibroblasts via the METTL3/miR-21/PTEN signaling pathway. Next, we will proceed to validate these findings in human LR-MSCs and lung tissues of PF patients. Moreover, METTL3-mediated m6A methylation play a significant role in regulation of cell function, and its downstream targets are flexible, with a wide range. Additional studies should be conducted in the future to explore these aspects in greater detail. Targeting METTL3-mediated m6A RNA methylation and its downstream targets may provide novel therapeutic strategies for the prevention and treatment of PF.

### Supplementary Information

**Additional file 1: Figure S1.** Construction of METTL3-overexpressing or METTL3-silenced LR-MSCs. **A**, **B** The mRNA and protein expression levels of METTL3 in METTL3-overexpressing or METTL3-silenced LR-MSCs compared to controls. β-Actin was used as a reference gene. **P < 0.01. **Figure S2.** Successful construction of a bleomycin-induced pulmonary fibrosis mouse model. **A** HE staining of lung tissues from mice with or without bleomycin exposure. **B** The α-SMA, type I collagen, and vimentin expression levels in lung tissues from mice with or without bleomycin exposure. β-Actin was used as a reference gene. **P < 0.01. **Figure S3.** Generation of the METTL3-silenced mouse model. **A**, **B** The mRNA and protein expression levels of METTL3 in the lung tissue of mice treated with METTL3 knockout adenovirus or control adenovirus. β-Actin was used as a reference gene. **P < 0.01. **Figure S4.** Local RNA structures of pri-miR-21 with very high confidence in m6A modification potential. Yellow, m6A binding sites. **Figure S5.** The construction of PTEN-overexpressing or PTEN-silenced LR-MSCs. **A**, **B** The mRNA and protein expression levels of PTEN in PTEN-overexpressing or PTEN-silenced LR-MSCs compared to controls. β-Actin was used as a reference gene. **P < 0.01.

**Additional file 2: Table S1.** Primers used in this study.

## Data Availability

No new datasets were generated during the current study.
