# Supplementary material for: METTL3-mediated m6A RNA methylation induces the differentiation of lung resident mesenchymal stem cells into myofibroblasts via the miR-21/PTEN pathway
Source: Respir Res. 2023 Nov 28;24:300. doi: 10.1186/s12931-023-02606-z (PMC10683095; doi:10.1186/s12931-023-02606-z)
Supplement: Supplementary file 1 — Additional file 1: Figure S1. Construction of METTL3-overexpressing or METTL3-silenced LR-MSCs. A, B The mRNA and protein expression levels of METTL3 in METTL3-overexpressing or METTL3-silenced LR-MSCs compared to controls. β-Actin was used as a reference gene. **P < 0.01. Figure S2. Successful construction of a bleomycin-induced pulmonary fibrosis mouse model. A HE staining of lung tissues from mice with or without bleomycin exposure. B The α-SMA, type I collagen, and vimentin expression levels in lung tissues from mice with or without bleomycin exposure. β-Actin was used as a reference gene. **P < 0.01. Figure S3. Generation of the METTL3-silenced mouse model. A, B The mRNA and protein expression levels of METTL3 in the lung tissue of mice treated with METTL3 knockout adenovirus or control adenovirus. β-Actin was used as a reference gene. **P < 0.01. Figure S4. Local RNA structures of pri-miR-21 with very high confidence in m6A modification potential. Yellow, m6A binding sites. Figure S5. The construction of PTEN-overexpressing or PTEN-silenced LR-MSCs. A, B The mRNA and protein expression levels of PTEN in PTEN-overexpressing or PTEN-silenced LR-MSCs compared to controls. β-Actin was used as a reference gene. **P < 0.01. [file 12931_2023_2606_MOESM1_ESM.pdf]

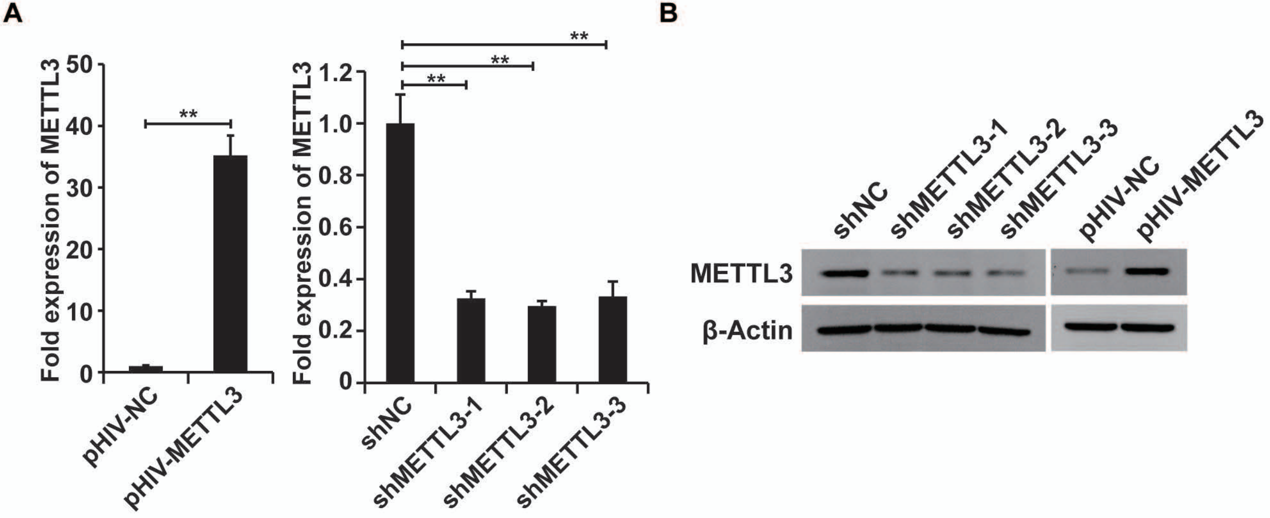
A
Fold expression of METTL3
50
40
30
20
10
0
**
pHIV-NC
pHIV-METTL3
Fold expression of METTL3
1.2
1.0
0.8
0.6
0.4
0.2
0
**
**
**
shNC
shMETTL3-1
shMETTL3-2
shMETTL3-3
B
shNC
shMETTL3-1
shMETTL3-2
shMETTL3-3
pHIV-NC
pHIV-METTL3
METTL3
β-Actin

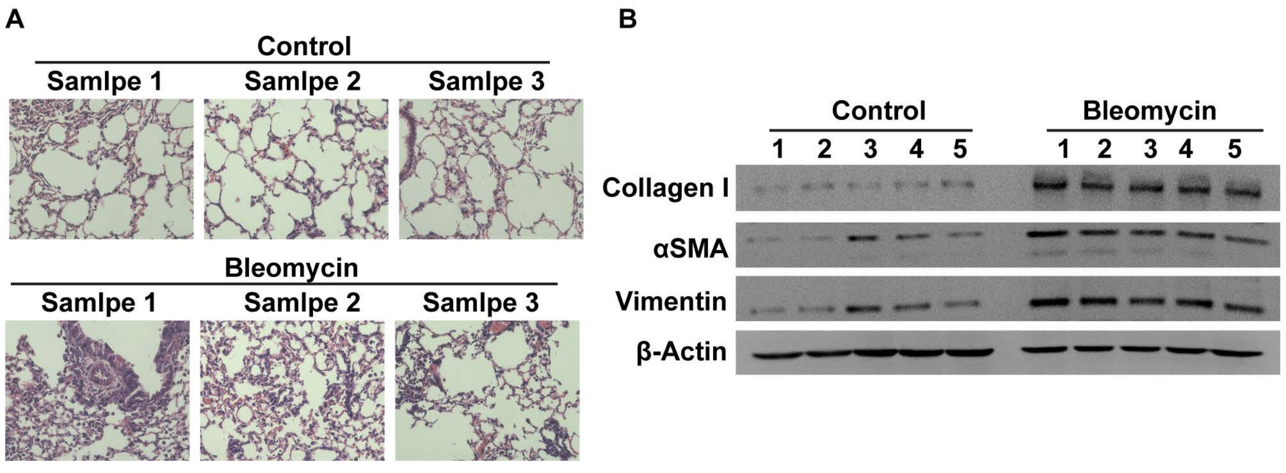
A
Control
Samlpe 1
Samlpe 2
Samlpe 3
Bleomycin
Samlpe 1
Samlpe 2
Samlpe 3
B
Control
Bleomycin
1
2
3
4
5
1
2
3
4
5
Collagen I
αSMA
Vimentin
β-Actin

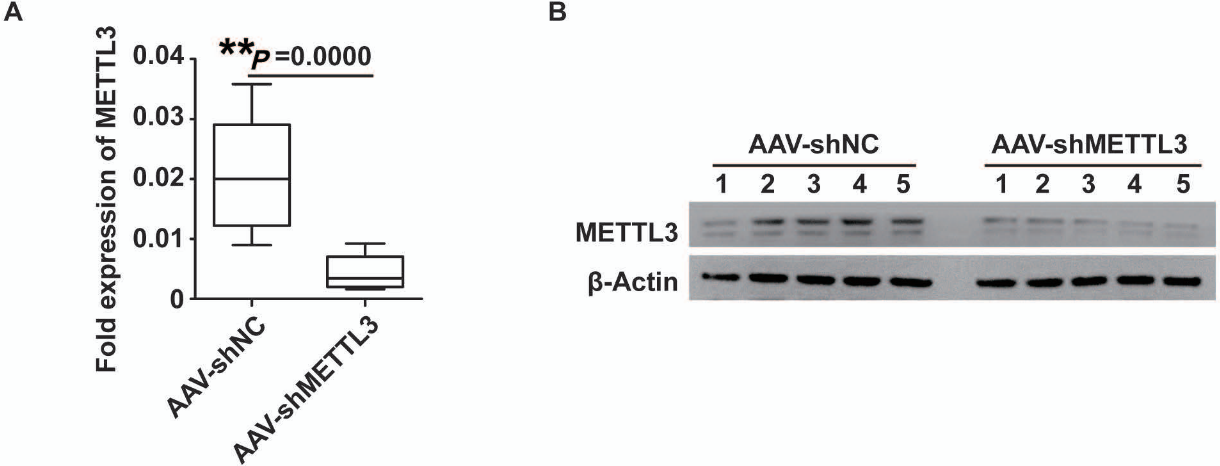
A
B
**P =0.0000
Fold expression of METTL3
0.04
0.03
0.02
0.01
0
AAV-shNC
AAV-shMETTL3
AAV-shNC
AAV-shMETTL3
1 2 3 4 5
1 2 3 4 5
METTL3
β-Actin

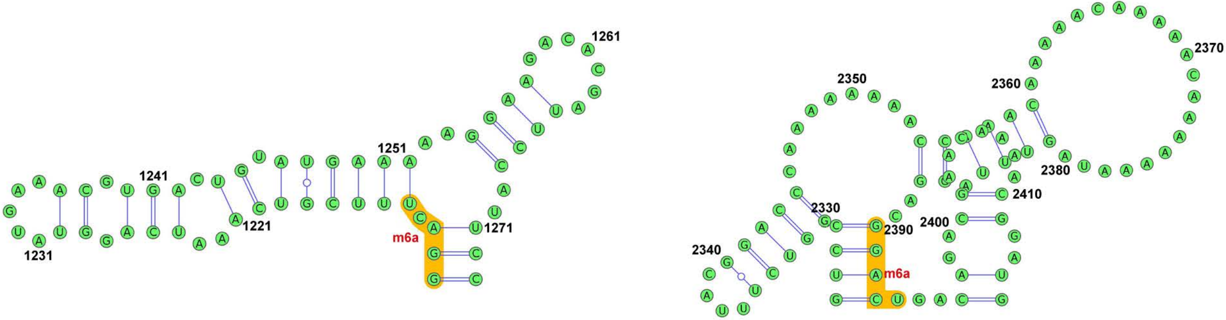
1221
1231
1241
1251
1261
1271
m6a
2330
2340
2350
2360
2370
2380
2390
2400
2410
m6a

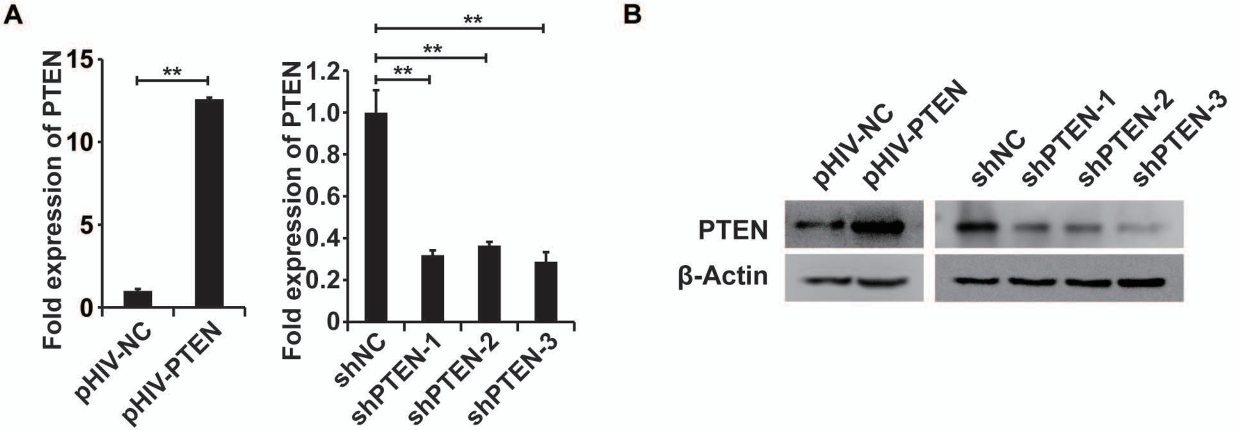
A
B
Fold expression of PTEN
15
10
5
0
**
pHIV-NC
pHIV-PTEN
Fold expression of PTEN
1.2
1.0
0.8
0.6
0.4
0.2
0
**
**
**
shNC
shPTEN-1
shPTEN-2
shPTEN-3
pHIV-NC
pHIV-PTEN
shNC
shPTEN-1
shPTEN-2
shPTEN-3
PTEN
β-Actin
