# Supplementary material for: METTL3-mediated m6A RNA methylation induces the differentiation of lung resident mesenchymal stem cells into myofibroblasts via the miR-21/PTEN pathway
Source: Respir Res. 2023 Nov 28;24:300. doi: 10.1186/s12931-023-02606-z (PMC10683095; doi:10.1186/s12931-023-02606-z)
Supplement: Supplementary file 2 — Additional file 2: Table S1. Primers used in this study. [file 12931_2023_2606_MOESM2_ESM.docx]

**Table S1. Primers used in this study.**

| **Genes** | **Sequences** |
| --- | --- |
| **METTL3** |  |
| Forward | 5'- TCCGGACGGCATCATCTCTA-3' |
| Reverse | 5'-TTAAGGTGTGGCCTGTAGCC -3' |
| **METTL14** |  |
| Forward | 5'-GTGGTCGGGAAAGAAACCGA -3' |
| Reverse | 5'-GCTCTGAAGCAAGTCTCCACT -3' |
| **WTAP** |  |
| Forward | 5'- CTGTCAGGGCACTCTTCCTC-3' |
| Reverse | 5'- CCTGGCAGTCCTAAACCCAC-3' |
| **FTO** |  |
| Forward | 5'- GTGCCAGGAGTAAGACCCAC-3' |
| Reverse | 5'-TGGCATTTTGGGTACCTCCC -3' |
| **ALKBH5** |  |
| Forward | 5'- TTGGTCACTGACACCCCTTG-3' |
| Reverse | 5'-GCAGCTTCTCTACCAAGCCA -3' |
| **PTEN** |  |
| Forward | 5'-TAACATGCAGGCTTCTGAGGG -3' |
| Reverse | 5'-AAAATCCACACACAAGCCACT -3' |
| **Collagen I** |  |
| Forward | 5'- CTCCTCCATTGCTCCCCAAAT-3' |
| Reverse | 5'- GGGTGCTGGGTAGGGAAGTA-3' |
| **αSMA** |  |
| Forward | 5'-CGCCTCCAGTTCCTTTCCAA -3' |
| Reverse | 5'-CTAGGCCAGGGCTACAAGTT -3' |
| **β-Actin** |  |
| Forward | 5'- TTACAGGAAGTCCCTCACCC-3' |
| Reverse | 5'- ACACAGAAGCAATGCTGTCAC-3' |
| **miR-21-5p** |  |
| Forward | 5'- CTCGGTAGCTTATCAGACTG-3' |
| RT | 5'-GTCGTATCCAGTGCAGGGTCCGAGGTATTCGCACTGGATACGACTCAACA -3' |
| **U6** |  |
| Forward | 5'-GTGATCACTCCCTGCCTGAG -3' |
| Reverse | 5'- GGACTTCACTGGACCAGACG-3' |
